# Sex Differences in Epidemiology and Risk Factors of Acute Coronary Syndrome in Chinese Patients with Type 2 Diabetes: A Long-Term Prospective Cohort Study

**DOI:** 10.1371/journal.pone.0122031

**Published:** 2015-04-01

**Authors:** Jian Gang Duan, Xiang Yan Chen, Li Wang, Alex Lau, Adrian Wong, G. Neil Thomas, Brian Tomlinson, Roxanna Liu, Juliana C. N. Chan, Thomas W. Leung, Vincent Mok, Ka Sing Wong

**Affiliations:** 1 Department of Emergency, Xuanwu Hospital, Capital Medical University, Beijing, China; 2 Department of Medicine and Therapeutics, Chinese University of Hong Kong, Prince of Wales Hospital, Shatin, Hong Kong Special Administrative Region; 3 Department of Psychological Studies and Center for Psychosocial Health and Aging, The Hong Kong Institute of Education, Hong Kong Special Administrative Region; 4 Department of Public Health, Epidemiology and Biostatistics, University of Birmingham, Birmingham, United Kingdom; National University of Singapore, SINGAPORE

## Abstract

**Objective:**

Diabetic patients with acute coronary syndrome (ACS) are at higher risk of poor outcome than are non-diabetic patients with ACS. Few studies have focused on sex-related ACS incidence, ACS-related mortality or risk factors to affects sex specific ACS in Chinese with Type 2 diabetes mellitus (T2DM). Based on a hospital-based cohort of Chinese patients with T2DM, we aimed to investigate whether there was sex difference in ACS or ACS-related mortality or risk factors of ACS.

**Methods:**

Totally 2,135 Hong Kong Chinese with T2DM were recruited during 1994-1996 and followed up until August 2012. We systematically analyzed sex-related ACS incidence and ACS-related mortality and risk factors with χ^2^-squared test, descriptive statistics and survival analysis.

**Results:**

Regular follow-up was completed in 2,105 subjects (98.6%), with a median period of 14.53 years. The occurrence of ACS was recorded among 414 patients (19.7%) and ACS-related death among 104 patients (4.9%). ACS incidences increased with age in both men and women, and men had a higher prevalence of ACS than women across different age categories and different follow-up periods (log rank χ^2^=20.32, P<0.001). The transition of ACS incidences from slow to rapid increase were about 5 years earlier in men (at 51-55 years) than in women (55-60 years). Among ACS patients, cumulative ACS-related mortalities was similar between men and women (log rank χ^2^=0.063, P=0.802). Besides age and albuminuria, different profiles of risk factors accounted for the occurrence of ACS between men and women.

**Conclusions:**

Our findings demonstrated sex differences in ACS incidence and risk factors, but not in ACS-related mortality in Chinese patients withT2DM. These findings suggest that screening and prevention campaigns should be optimized for men and women, which may help to identify diabetic patients at higher risk of coronary heart disease.

## Introduction

Despite major breakthroughs in management, cardiovascular disease (CVD)—and more specifically acute coronary syndromes (ACS)—remains a leading cause of mortality worldwide [1], [2]. There is a strong association between diabetes and CVD mortality, poor clinical outcome and morbidity [3]. A meta-analysis including almost 700,000 subjects with no history of myocardial infarction (MI), angina or stroke at baseline, revealed that diabetes confers an approximately twofold excess risk for coronary heart disease (CHD), major stroke and deaths attributed to other vascular causes [4]. ACS is even more insidious because diabetic patients with this condition often show only mild or atypical symptoms and signs, and many suffer silent myocardial ischemia and infarction [5]. According to the literature, diabetic patients are still at increased residual risk of long-term mortality despite the overall improvement in the management and outcomes of the ACS [6].

Besides diabetes, numerous and remarkably consistent studies have reported sex differences in ACS in major registries and clinical trials in Western populations[7], [8]. Many ACS studies have shown women to be older and with higher incidences of comorbidities at presentation [8], [9]. In Asian populations, sex differences in the demographic characteristics, risk factors, treatments, and outcomes of ACS was demonstrated in a study in Malaysia [10].

According to the National Diabetes Prevalence Survey in China from 2007 to 2008, the prevalence of diabetes in Chinese adults was 9.7% [11], which will consequently create a heavy burden of diabetes and its complications in the aging population in the near future. Considering the increased burden of type 2 diabetes mellitus (T2DM) on ACS [12] and its potential sex difference in its distribution and effects on ACS, we aimed to explore the occurrence of ACS and ACS-related mortality in a hospital-based cohort of Chinese patients with T2DM.

## Materials and Methods

### Study population

The study protocol was approved by the Clinical Research Ethics Committee of the Chinese University of Hong Kong. In this prospective cohort study, individuals with T2DM were recruited from the Diabetes Clinic at the Prince of Wales Hospital between 1994 and 1996. Individuals gave written, informed consent to participate in the study. All participants were Hong Kong Chinese by birth. Participants with history of stroke, transient ischemic attack or ischemic heart disease were excluded.

Participants were considered to have T2DM if fasting plasma glucose was ≥ 7.8 mmol/l, if 2-h plasma glucose was ≥11.1 mmol/l after a 75-g oral glucose tolerance test, or if they were taking antidiabetic medication at the time of study enrollment [13]. Participants with type 1 diabetes [14] accounted for a small percentage (1.7%) of the individuals approached for enrollment, and they were excluded from the study.

### Baseline Assessment

Upon enrollment, patients were examined by transcranial Doppler (TCD) using an EME TC-2000 scanner. The middle cerebral artery (MCA) was analyzed for the presence of stenosis using standard criteria [15]. All TCD evaluations were performed by two experienced operators (K.S.W., R.L.) using a standardized protocol.

A questionnaire including information on demographic characteristics, medical history (including oral hypoglycemic agents and insulin, angiotensin-converting enzyme inhibitors (ACEI), aspirin, and statin), and lifestyle factors was administered by trained interviewers. Smoking was defined as having smoked 100 cigarettes in one’s lifetime [16]. Waist circumference was measured on standing participants midway between the lower edge of the costal arch and the upper edge of the iliac crest [16]. Subjects were defined as hypertensive if, after 5 min rest, their seated systolic blood pressure (SBP) was ≥ 140 mm Hg and/or diastolic blood pressure (DBP) ≥ 90 mm Hg on at least two occasions, or if they were receiving blood pressure-lowering medication [17]. Patients were assessed to rule out secondary causes of hypertension and renal disease. Several biochemical parameters were measured after overnight fasting, including lipid and glycemic profiles in plasma and the albumin-to-creatinine ratio (ACR) in urine. Albuminuria was diagnosed when ACR was ≥3.5 mg/mmol [18] at enrollment. Peripheral artery disease (PAD) was diagnosed if claudication, gangrene, or ischemia-related amputation were present. Retinopathy was assessed by an ophthalmologist at enrollment. Fundi were examined through dilated pupils, and retinopathy was considered to be present if one or more of the following were detected: hemorrhage, microaneurysm, cotton wool spots, and/or laser coagulation scars related to diabetic retinopathy.

### Follow-up

Participants were followed up from the time of enrollment until August 2012. The primary end point was the first ACS event. An ACS event was defined as unstable angina or myocardial infarction, regardless of whether the infarction occurred with ST segment elevation or not. Secondary end point was ACS-related mortalities. Deaths due to ACS were identified based on international classification of disease-9 (ICD-9) discharge codes of 432–438, 410, or 413. The exact cause of death was determined from death certificates or medical records. When end points for a given participant were uncertain, we also reviewed paper-based medical records or called the patients or their relatives for follow-up.

Hospitalization and outcomes of study participants were tracked through the Hong Kong Hospital Authority Central Computer System, which records admissions to, and discharges from, all public hospitals in Hong Kong. Participants were unambiguously identified in this computer system by their unique Hong Kong Identity Card number, which is compulsory for all residents of Hong Kong.

### Statistical Analysis

Values of continuous variables are presented as mean ± SD; values for categorical variables, as percentages. Follow-up period were finished within a median period of 14.5 years (range: 0.3–19.59 years). Levels of hemoglobin A1c (HbA1c) are reported in terms of %. Baseline characteristics were compared separately between men and women using Χ^2^-squared and independent-samples *t* tests as appropriate.

Differences in ACS incidence and ACS-related mortality between men and women were compared using the Χ^2^-squared test. Differences in ACS incidence per 1000 person-years between men and women were analyzed by descriptive statistics for predefined age groups (yr): ≤35, 36–40, 41–45, 46–50, 51–55, 56–60, 61–65, 66–70, and ≥71. We used regression techniques to plot the relationship between age (x, independent variable) and ACS incidence (y, dependent variable) using a polynomial (quadratic) equation y = a+bx+cx^2^ [19]. Mean values of each-year ACS incidence and each-5-year ACS incidence were estimated separately for men and women using life-table analysis. Cumulative occurrences of ACS between men and women were assessed using Kaplan-Meier survival analysis. Cumulative ACS-related mortality was estimated separately in men and women with ACS using Kaplan-Meier survival analysis.

Cox proportional hazard regression models were performed firstly in the whole recruited subjects by including 13 variables: sex, age, smoking, diabetes duration, SBP, PAD, retinopathy, MCA stenosis, total cholesterol (TC), low-density lipoprotein (LDL), high-density lipoprotein (HDL), HbA1c and albuminuria. Then the Cox regression was repeated in men and in women excluding the variable of sex. Results are expressed as hazard ratios (HRs) with associated 95% confidence intervals (CIs).

For all statistical tests, two-sided P-values of <0.05 were considered statistically significant. All statistical analyses were performed in PASW Statistics (version 18.0, IBM, Chicago, USA).

## Results

A total of 2,197 individuals with T2DM were recruited between 1994 and 1996. After excluding 62 patients with ischemic heart disease at baseline, 2,135 patients with T2DM were recruited, with the mean age of 55.2±11.3 years old (range, 29–81) and men accounting for 41.0%.

### Comparisons of baseline characteristics between men and women

Table 1 demonstrated the comparison of baseline characteristics between men and women. Compared to women, men were younger and had lower level of SBP, higher level of DBP, lower level of HDL, a dramatically higher rate of smoking, shorter duration of T2DM but a higher rate of albuminuria at baseline. The prevalence of vascular events, including periphery artery disease, retinopathy and middle cerebral artery stenosis, was similar between men and women. Usage of anti-diabetic, anti-hypertension, aspirin and statin was also comparable between men and women.

**Table 1:** Comparisons of basic characteristics and risk factors between men and women in a cohort of Chinese patients with type 2 diabetes.

Characteristic	All (N = 2135)	Men (n = 876)	Women (n = 1259)	P-value
**Age (year)**	55.18±11.31	54.61±11.24	55.59±11.35	0.048
**SBP (mmHg)**	138.18±21.44	136.07±20.64	139.65±21.86	<0.001
**DBP (mmHg)**	78.86±11.34	79.75±11.49	78.24±11.20	0.003
**TC (mmol/l)**	5.62±1.11	5.48±1.13	5.71±1.10	<0.001
**TG (mmol/l)**	1.78±1.64	1.81±1.61	1.76±1.66	0.488
**LDL (mmol/l)**	3.59±0.92	3.55±0.88	3.61±0.94	0.191
**HDL (mmol/l)**	1.26±0.36	1.16±0.31	1.34±0.38	<0.001
**HbA1c, %**	7.85±1.87	7.91±1.90	7.80±1.84	0.191
***Risk factors***				
**Smoking**	575 (26.9)	493 (56.3)	82 (6.5)	<0.001
**Diabetes duration (mo.)**	80.71±72.79	71.80±67.86	86.90±75.45	<0.001
**Albuminuria**	624 (29.2)	276 (32.5)	348 (28.4)	0.047
**PAD**	42 (2.0)	17 (2.0)	25 (2.0)	0.993
**Retinopathy**	484 (22.7)	201 (22.9)	283 (22.5)	0.815
**MCA stenosis**	256 (12)	98 (11.2)	158 (12.5)	0.340
***Medications***				
**Anti-diabetic**	1846 (86.5)	770 (88.0)	1076 (85.7)	0.120
**ACEI**	250 (11.7)	103 (11.8)	147 (11.7)	0.962
**Aspirin**	15 (0.7)	6 (0.7)	9 (0.7)	0.933
**Statin**	94(4.4)	36(4.1)	58(4.6)	0.578

Values are expressed as the mean ±SD or n (%).

Note: SBP, systolic blood pressure; DBP, diastolic blood pressure; TC, total cholesterol; TG, triglyceride; LDL, low-density lipoprotein; HDL, high-density lipoprotein; HbA1c, hemoglobin A1c; PAD, peripheral artery disease; MCA, middle cerebral artery; ACEI, angiotensin-converting enzyme inhibitors.

### ACS incidence in men and women

**Fig 1:**
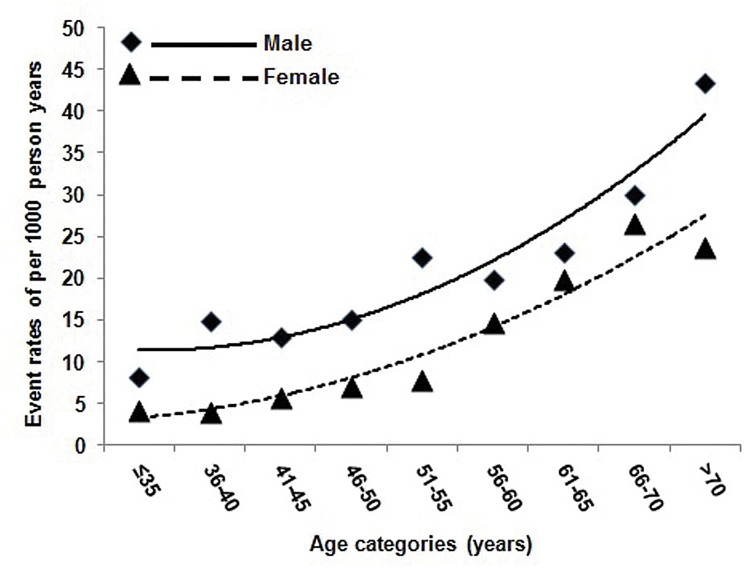
ACS incidence per 1000 person-years among men and women with in different age categories. ACS incidence generally increased with age among (A) 876 men and (B) 1259 women. Descriptive analyses are displayed with best-fit curves defined by the polynomial equations (A) y = 0.4568x^2^ -1.0512x +11.96 (R^2^ = 0.9074) and (B) y = 0.2867x^2^ + 0.1611x + 2.8626 (R^2^ = 0.9261).

**Fig 2:**
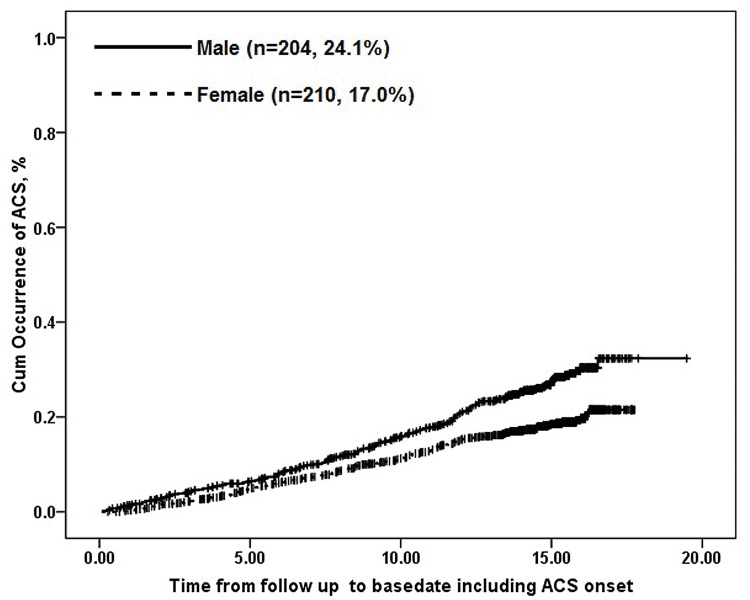
Survival plots of examining the association between sex and occurrence of ACS. The continuous line depicts data for 204 men; the dashed line depicts data for 210 women. Cumulative occurrences of ACS were higher among men than women over time (log rank Χ^2^ = 20.32, P<0.001).

During the follow-up of a median year of 14.5 years, the incidence of ACS was recorded in 414 patients (19.7%). Men had a higher incidence of ACS than women (24.1% vs. 17.0%, p<0.001). By categorizing the patients of men and women into different age groups (every 5 years from 35 years old), the ACS incidences per 1000 person-years increased smoothly with age in both men and women and the incidences were consistently higher in men than those in women for corresponding age groups and the transition from slow to rapid increase occurred earlier in men (at 51–55 yr) than in women (56–60 yr), the changing trends of which was represented in Fig. 1. Each-year ACS incidences and each-5-year ACS incidences were calculated respectively across different age categories in table 2, which were in accordance with the above analysis. Cumulative incidences of ACS in men was higher than in women (log rank Χ^2^ = 20.32, P<0.001) (Fig. 2).

**Table 2:** Each-year and each-5-year incidence (%) of acute coronary syndrome among Chinese adults with type 2 diabetes, stratified by sex and age from 35 years old.

Age range, yr	Each-year incidence (%)		Each-5-year incidence (%)	
	Men	Women	Men	Women
**≤35**	0.90	0.50	5.25	2.25
**36–40**	1.61	0.44	7.50	2.00
**41–45**	1.33	0.67	7.25	2.75
**46–50**	1.44	0.56	6.5	3
**51–55**	2.33	0.78	11.50	3.75
**56–60**	1.56	1.83	7.50	7.25
**61–65**	2.06	1.89	9.00	8.75
**66–70**	3.78	3.44	16.00	12.75
**>70**	3.94	2.22	16.25	9.75

### ACS-related mortality in men and women

**Fig 3:**
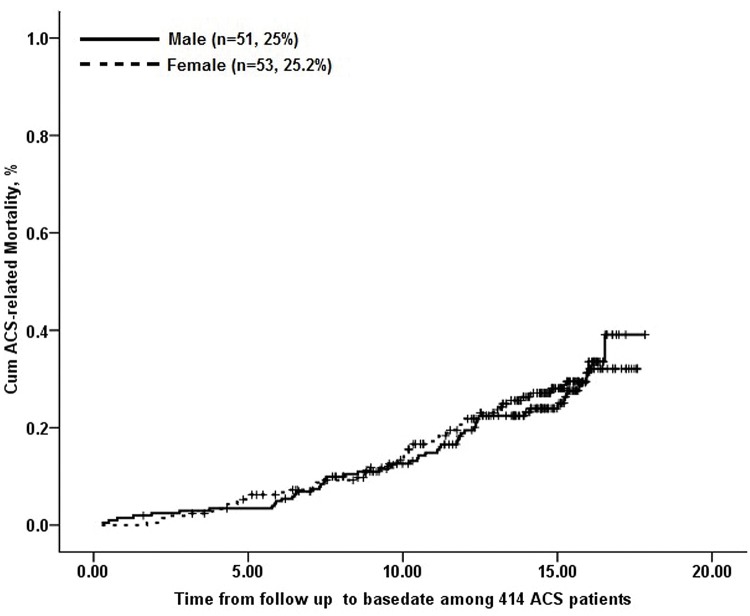
Kaplan-Meier survival curve for ACS-related death in 414 Chinese type 2 diabetic subjects with ACS. The continuous line depicts data for 51 men; the dashed line depicts data for 53 women. No significant differences exist between ACS-related mortalities in men and women (log rank Χ^2^ = 0.063, P = 0.802).

Among the 414 ACS patients, 104 patients died during the period of follow up (4.9%). The incidence of ACS-related death was comparable between men and women (5.8% vs. 4.2%, p = 0.089).Cumulative ACS-related mortalities were also similar different between men and women (log rank Χ^2^ = 0.063, P = 0.802; Fig. 3).

### Sex differences in risk factors of ACS (table 3)

The first Cox regression model in the whole subjects with T2DM found that age (1.029, 95%CI: 1.018–1.041), male (1.774, 95%CI: 1.418–2.218), diabetes duration at baseline (1.004, 95%CI: 1.002–1.005), SBP (1.007, 95%CI: 1.007–1.013), PAD (4.181, 95%CI: 2.661–6.694), TC (1.295, 95%CI: 1.165–1.440), HDL (0.559, 95%CI: 0.396–0.790) and albuminuria(1.619, 95%CI: 1.298–2.034) were independent predictors of ACS.

**Table 3:** Cox proportional hazard regression models to identify predictors of acute coronary syndrome among Chinese men and women with type 2 diabetes mellitus after adjusting for different confounders.

Variable	All			Men			Women		
	**HR**	**95%CI**	**P value**	**HR**	**95%CI**	**P value**	**HR**	**95%CI**	**P value**
**Male**	1.774	1.418–2.218	<0.001	------	------	------	------	------	------
**Age(year)**	1.029	1.018–1.041	<0.001	1.022	1.006–1.038	0.006	1.047	1.029–1.065	<0.001
**Smoking**	1.269	0.969–1.661	0.083	1.067	0.768–1.482	0.698	1.895	1.163–3.087	0.01
**Diabetes duration (mo.)**	1.004	1.002–1.005	<0.001	1.004	1.002–1.006	<0.001	1.001	0.999–1.003	0.285
**SBP(mmHg)**	1.007	1.002–1.013	0.007	1.003	0.994–1.012	0.533	1.009	1.002–1.017	0.015
**PAD**	4.181	2.611–6.694	<0.001	1.916	0.766–4.793	0.165	9.789	5.299–18.082	<0.001
**Retinopathy**	1.214	0.942–1.565	0.134	1.264	0.845–1.890	0.254	1.391	0.958–2.019	0.083
**MCA stenosis**	1.163	0.868–1.558	0.313	1.064	0.624–1.816	0.819	1.541	0.988–2.404	0.057
**TC (mmol/l)**	1.295	1.165–1.440	<0.001	1.046	0.639–1.712	0.858	1.09	0.929–1.279	0.288
**LDL (mmol/l)**	0.953	0.685–1.326	0.775	1.541	1.285–1.849	<0.001	0.9	0.541–1.496	0.685
**HDL (mmol/l)**	0.559	0.396–0.790	0.001	0.483	0.269–0.867	0.015	0.801	0.492–1.305	0.373
**HbA1c, %**	1.029	0.970–1.091	0.343	0.965	0.677–1.377	0.846	1.715	1.172–2.511	0.006
**Albuminuria**	1.619	1.289–2.034	<0.001	1.731	1.235–2.427	0.001	1.993	1.407–2.824	<0.001

CI, confidence interval; HR, hazard ratio; SBP, systolic blood pressure; PAD, peripheral artery disease; MCA, middle cerebral artery; TC, total cholesterol; LDL, low-density lipoprotein; HDL, high-density lipoprotein; HbA1c, hemoglobin A1c.

The second Cox regression model in men showed that age (1.022, 95%CI: 1.006–1.038), diabetes duration at baseline (1.004, 95%CI: 1.002–1.006), LDL (1.541, 95%CI: 1.285–1.849), HDL (0.483, 95%CI: 0.296–0.867) and albuminuria(1.731, 95%CI: 1.235–2.427) were independent predictors of ACS.

The third model in women found that age (1.047, 95%CI: 1.029–1.065), smoking (1.895, 95%CI: 1.163–3.087), SBP (1.009, 95%CI: 1.002–1.017), PAD (9.798, 95%CI: 5.299–18.082), HbA1c (1.715, 95%CI: 1.172–2.511) and albuminuria(1. 933, 95%CI: 1.407–2.824) were independent predictors of ACS. There was a trend that MCA stenosis (1.541, 95%CI: 0.988–2.404) was an independent risk factors of ACS in women.

## Discussion

In this hospital-based cohort, the prevalence of ACS increases with age in both men and women in Chinese patients with T2DM. Our findings support male predominance of ACS occurrence but similar ACS-related mortality between men and women in patients with T2DM. The transition of ACS incidences from slow to rapid increase were about 5 years earlier in men (at 51–55 years) than in women (55–60 years). Age and albuminuria are verified to be risk factors of ACS in both men and women, but other risk factors vary between men and women.

Some studies have suggested sex differences in presentation and treatment of ACS, but there are many uncertainties and discrepancies between these studies [20], [21]. CHD affects men more than women in the general population [22], [23], which is consistent with male predominance of ACS in Chinese diabetic patients. The rate of ACS in this cohort was as high as 19.7%, which is higher than the rate of ACS in those without diabetes whether in the western population or in Chinese population [23], [24]. Claassen et al also reported that coronary vascular disease in women develops 7 to 10 years later than in men, potentially because of a protective effect of estrogens [22]. Epidemiological studies, including the Framingham study, showed that CHD presents at an earlier age in men than in women [25], [26]. Therefore, younger women benefit from the protective effects of endogenous estrogens, including estradiol [27], which may inhibit age-related vascular remodeling [27], such as vascular smooth muscle cell proliferation and endothelial dysfunction. Other studies demonstrated that estradiol lowers cholesterol levels and improves the vascular tone [28], [29]. Therefore, the male prevalence of CHD and the gap between men and women begin to narrow after menopause [22], which has been recognized as a risk factor for ACS due to the reduction in endogenous estrogen [30].

Most studies suggest that diabetes is a stronger risk factor of CHD for women than men [31], but few have adjusted their results for classic risk factors: age, hypertension, total cholesterol level, and smoking [32], [33]. However, by performing a meta-analysis including 16 studies, Kanaya et al reported that the excess relative risk of coronary heart disease mortality in women vs men with diabetes was absent after adjusting for classic risk factors [32]. Similar to the above findings, ACS-related mortality was similar between men and women in our cohort of Chinese patients with T2DM. Maas also reported that coronary vascular disease is the main cause of death among women and its occurrence narrows women’s survival advantage over men [34]. Cabrerizo-Garcia, et al. interpreted that ACS-related worse prognosis in women than in men was due to their unfavorable baseline characteristics rather than due to sex difference [35]. Due to the complexity of different risk factors, many controversies still exist regarding sex difference in ACS among patients with T2DM. Age, smoking, and other baseline characteristics are required to be taken into account when interpreting the effects of diabetes on the prevalence of ACS or ACS-related mortality.

Smoking, diabetes, hypercholesterolaemia and hypertension are well established risk factors for the development of coronary artery disease [36], which presents different features in men and women. Among our cohort of Chinese diabetic patients, age, male, diabetes duration at baseline, SBP, PAD, TC, HDL and albuminuria were independent predictors of ACS; age, diabetes duration at baseline, LDL, low HDL and albuminuria were demonstrated to be predictors of ACS in men and age, smoking, SBP, PAD, HbA1c and albuminuria in women. Although most of the risk factors have been well established [18], [23], the different profiles of risk factors reflect the variations of clinical features and mechanisms of ACS between men and women with diabetes. Within this cohort of diabetic patients, our previous study [18] reported that albuminuria was associated with increased insulin resistance and adverse lipid profiles, which may explain the reason why albuminuria is consistently found to be a predictor among the whole diabetic population, men or women. The identification of different predictors between men and women may provide potential targets for treatment to slow the progression of disease and to reduce the risk of ACS in individual diabetic patients.

The main strengths of this study include its prospective design, large sample size and long follow-up, which provides valuable demographic variable and traditional risk factors to explore the sex-related predictors for cardiovascular disease and mortality in patients with diabetes. Nevertheless, the study does have some important limitations. First, smoking was not found to be an independent factor related to ACS incidence in male diabetic patients. Based our database, we only recorded the proportion of smoking (defined as having smoked 100 cigarettes in one’s lifetime), and included this variable into regression model. Unfortunately, we did not collect the information to categorize smoking habits to current or ever smoking, which may provide more evidence about smoking effects on ACS. Further studies are required to explore the effects of smoking intensity and duration on ACS in diabetic patients. Secondly, the details of drugs treatments (anti-diabetic medication, antiplatelet therapy, anti-hypertensive medicine and statin therapy) were not collected properly in this large-sized cohort, especially the duration and dose of specific medication. Considering that the duration of drug treatments may influence the treatment effects, the confounding effects of medication was not properly adjusted with the present design.

In conclusion, ACS incidence increase with age in both men and women with T2DM. Male predominance of ACS in Chinese patients with T2DM reflects the sex-difference in the risk of CHD. Similar ACS-related mortality between men and women may be interpreted due to the effects of other risk factors accounting for mortality in this population. Further studies are required to investigate the sex-dependent mechanisms of CHD, which may improve the clinical strategies for prevention and treatment of diabetic patients.
